# Variability in somatic embryo-forming capacity of spinach

**DOI:** 10.1038/s41598-020-76279-9

**Published:** 2020-11-09

**Authors:** Maja Belić, Snežana Zdravković-Korać, Branka Uzelac, Dušica Ćalić, Suzana Pavlović, Jelena Milojević

**Affiliations:** 1 Department of Plant Physiology, Institute for Biological Research “Siniša Stanković” - National Institute of Republic of Serbia, University of Belgrade, Bulevar despota Stefana 142, 11 060 Belgrade, Serbia; 2 Institute for Vegetable Crops, Karađorđeva 71, 11420 Smederevska Palanka, Serbia

**Keywords:** Plant biotechnology, Plant sciences

## Abstract

High variability in somatic embryo (SE)-forming capacity has previously been observed in several spinach cultivars. Such variability frequently accounted for more variation in embryogenic response of the explants than the factor being investigated. Hence, the variability in embryogenic capacity was examined in the present study at both the population and the single-seedling level, using seeds of spinach cultivar Matador obtained from nine European seed companies. Seed population obtained from Slovenia (Sl) was superior to others, with the highest regeneration frequency (100%) and the highest mean SE number (14.4). A total of 82% of these seedlings had 80–100% of regenerating explants, while in populations with intermediate embryogenic capacity approximately 40% of seedlings had 20–60% of regenerating explants. The explants from the majority of seedlings (52–100%) in the least responsive populations were irresponsive. Furthermore, the explants from Sl seedlings regenerated from 10–20 (43.5%) up to > 20 (27.6%) SEs on average, while the explants from the majority of seedlings belonging to other populations regenerated 1–10 SEs. The present study strongly indicates that the variability of plant material must not be overlooked, because choosing more responsive individuals for one treatment and less responsive ones for another may lead to misinterpretation of the data.

## Introduction

Spinach (*Spinacia oleracea* L.) is an economically important, green leafy vegetable, used either fresh or processed in a human diet. Spinach leaves are highly nutritious, rich in vitamins, minerals, phytochemicals and dietary fibbers, thus rendering a spinach-supplemented diet numerous health-promoting effects^1–4^. For this reason, spinach has become increasingly popular over the past several decades, making a tenfold increase in the production, according to the Food and Agriculture Organization (FAO) of the United Nations (https://faostat3.fao.org/browse/Q/QC/E). It is cultivated worldwide in the temperate regions, in more than 50 countries.

To date, numerous spinach cultivars and hybrids are available^5–7^. However, conventional breeding is a slow process, especially in plant species with complex sex determination, such as spinach^8^. Genetic engineering approach is a promising alternative, but it demands an efficient and reproducible regeneration system. Regeneration by somatic embryogenesis was achieved in spinach at the end of the twentieth century^9–13^. However, it is still recalcitrant to in vitro regeneration; hence high variability in regeneration capacity of numerous spinach cultivars has been reported^10,14,15^, most likely as a result of individual variability of this trait within the seed populations tested^16,17^. Accordingly, high variations in the regeneration response of individuals within ecotypes, accessions, and even inbred lines have also been reported in numerous plant species^18–20^.

In our research on somatic embryogenesis in spinach, random sampling of individuals for different treatments frequently produced inconclusive results, because individual variability accounted for more variation in embryogenic response of the explants than a factor being investigated (e.g. light or gibberellin impact on somatic embryo induction from the root explants). Such variabilities in embryogenic response are presumably caused by genetic and other factors affecting the physiological status of the plant donor material. Thus, annulling the variability, e. g. by using genetically uniform plant material grown under the same conditions for all treatments^21^ or by adopting an appropriate sampling method, appears to be a prerequisite for obtaining reliable results.

It was shown that two spinach ribosome-inactivating proteins, SoRIP1 and SoRIP2, were differentially expressed during somatic embryo induction and regeneration^22,23^. Our previous study showed that *SoRIP2* expression, which peaked in somatic embryos at the globular stage of development, could be used as a reliable marker for the assessment of regeneration capacity of the explants^24^.

Therefore, the objective of the present study was to evaluate the importance of inter- and intra-population variability in somatic embryo regeneration capacity of spinach root explants and to establish a method for sampling of plant material, to assure the reliability and reproducibility of the obtained results. For experimentation in the present study, seeds of spinach cultivar Matador that is widely grown in most of the European countries were obtained from nine European seed companies located in Slovenia (Sl), Poland (P), Serbia (Sr), England (E), Germany (G), Lithuania (L), Ukraine (U), Russia (R) and Italy (I). In order to test the sampling method of plant material for induction of somatic embryogenesis, expression of *SoRIP2* marker gene and conventional somatic embryos quantification were used.

## Results

The nine populations tested in the present study greatly differed in terms of seed germinability, seedling development and somatic embryo (SE) regeneration capacity. Among them, L seeds germinated and converted into plantlets very quickly (Supplementary Table S1). As many as 64.4% of L seeds germinated within a week, and attained 100% germination within 3 weeks of cultivation. By contrast, only 3.7% and 5.9% of I and Sl seeds, respectively, germinated after a week, reaching only 53% and 64.4% germinability after 4 weeks. The majority of seed populations (E, G, R and U) attained approximately 71–74% germinability, while P and Sr seeds reached 87% and 90% germinability, respectively, over a 4-week cultivation period.

The root explants were taken from plantlets at 5-leaf stage of development. Seedlings derived from seeds obtained from the nine populations required variable periods of time to reach this stage of development; for the majority of populations (Sr, E, U, L, R and I) 22–27 days after sowing (DAS), and 32–35 DAS for G, P and Sl seedlings (Supplementary Table S1).

According to nested ANOVA, inter- and intra-population differences in embryogenic response were statistically significant (Supplementary Table S2). The frequency of seedlings regenerating SEs was strongly affected by both population (Pη^2^ = 0.782, p < 0.01) and individual seedling (Pη^2^ = 0.685, p < 0.01), while population and individuals had weaker effect on the mean SE numbers (Pη^2^ = 0.499, p < 0.01 and Pη^2^ = 0.376, p < 0.01, respectively), but quite strong effect on the SE-forming capacity (SEFC) indices (Pη^2^ = 0.732, p < 0.01 and Pη^2^ = 0.688, p < 0.01, respectively).

### Regeneration capacity at the population level

The highest frequencies of SE regeneration and their lowest intra-population variability were attained within Sl population, while in other populations rather high intra-population variabilities were observed, except for R and I populations, which did not regenerate SEs at all (Supplementary Fig. S1). However, for the mean SE numbers and the SEFC indices, lower intra- and inter-population variabilities were observed, except for Sl population, which showed the greatest values, but also greater variations than other seed populations (Supplementary Fig. S1).

**Figure 1:**
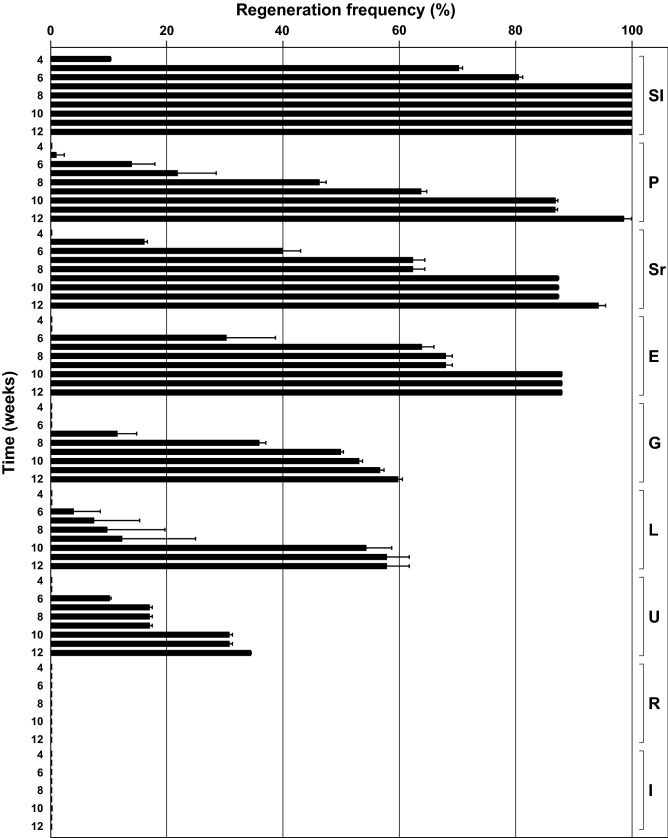
Regeneration frequencies of the root explants assessed over a 12-week cultivation time. The explants were taken from seedlings of spinach cv. Matador, derived from seeds obtained from nine European seed companies located in: Slovenia (Sl), Poland (P), Serbia (Sr), England (E), Germany (G), Lithuania (L), Ukraine (U), Russia (R) and Italy (I). Data represent the mean percentage ± standard error of responsive seedlings per population. A seedling was considered responsive if at least one root explant regenerated somatic embryos. Fifteen to twenty root explants were taken from each seedling, and 24–30 seedlings were assessed per population.

The explants of Sl seedlings regenerated first; 10.3% of these seedlings started regenerating SEs already in the 4th week of cultivation, while 70% and 100% of the seedlings responded within the 5th and the 7th week of cultivation, respectively (Fig. 1). The explants of other populations started regenerating later: P and Sr in the 5th, G, E, L, and U in the 6th week of cultivation, while R and I root explants did not regenerate until the end of the experiment (Fig. 1). Accordingly, seedlings of less responsive populations reached their highest frequencies of regeneration much later than Sl seedlings, attaining 50% responsiveness from 6th (Sr) through 7th (E), 9th (P and G) to 10th (L) week of cultivation.

**Figure 2:**
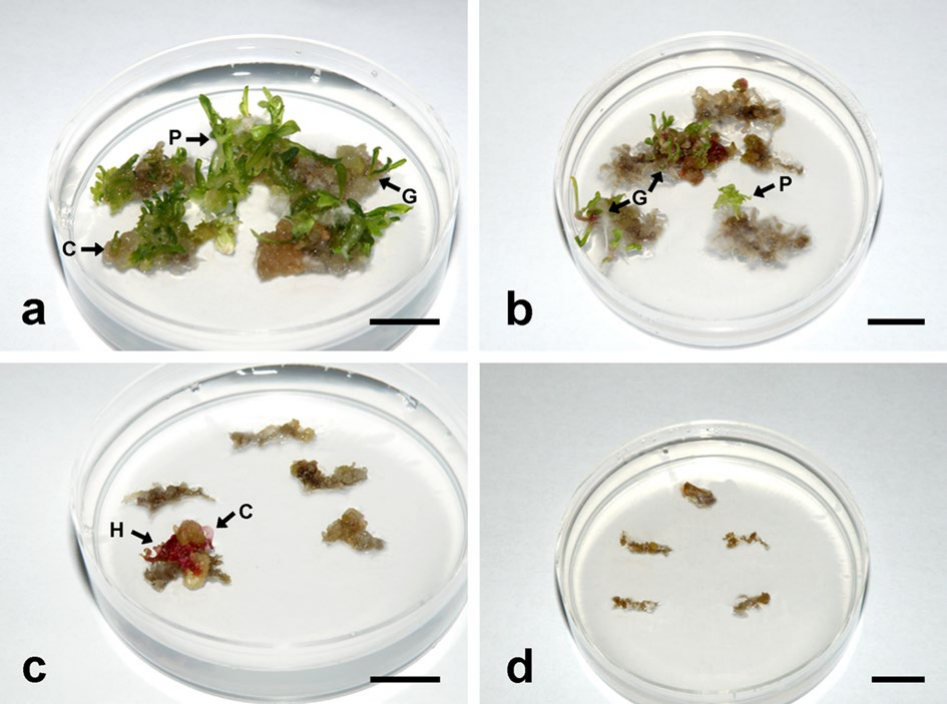
Variable embryogenic response of the root explants isolated from spinach seedlings derived from seeds obtained from: (**a**) Sl, (**b**) P, (**c**) U and (**d**) R populations, after 8 weeks of cultivation on IM. *H* heart-shaped somatic embryo, *C* cotyledonary-stage somatic embryo, *G* germinated somatic embryo, *P* somatic embryo-derived plant. Bars = 1 cm.

The explants taken from Sl seedlings exhibited the highest frequency of seedling responsiveness (100%), followed by P (98%), Sr and E (88%, each), G (60%), L (58%), U (34%), R and I (0%, each) population during a 12-week period of cultivation (Figs. 1, 2).

**Figure 3:**
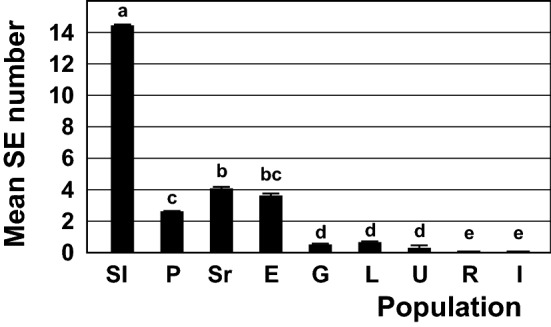
The mean somatic embryo number per explant regenerated from the explants cultivated on IM for 12 weeks. Seeds of spinach cultivar Matador were obtained from nine European seed companies located in: Slovenia (Sl), Poland (P), Serbia (Sr), England (E), Germany (G), Lithuania (L), Ukraine (U), Russia (R) and Italy (I). Fifteen to twenty root explants were taken from each seedling, and 24–30 seedlings were assessed per population. Data represent the mean value ± standard error, calculated for the whole population. Data denoted with different letters are statistically significant according Fisher’s LSD post hoc test for P ≤ 0.05.

Based on the mean SE number per explant, calculated for the whole population, the populations could be divided into four groups: (1) Sl population with the highest mean SE number (14.4 SEs on average per explant), (2) P, Sr and E populations with 2.6–4.1 SEs per explant, (3) G, L and U populations with only 0.3–0.6 SEs per explant, and (4) R and I populations without embryogenic response (Figs. 2, 3). Thus, the populations with greater number of responsive seedlings yielded greater number of SEs in the present study for shorter period of time than the populations with lesser number of responsive seedlings.

### Regeneration capacity at the individual seedling level

**Figure 4:**
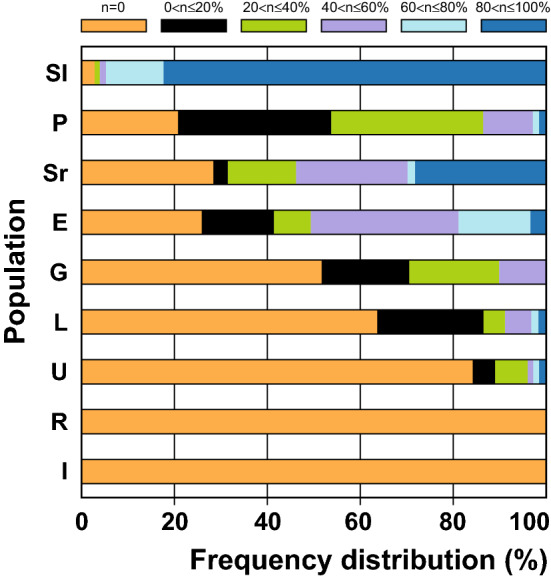
Frequency distribution of the proportion of seedlings belonging to six classes of the regeneration frequencies for nine populations of spinach cultivar Matador from Slovenia (Sl), Poland (P), Serbia (Sr), England (E), Germany (G), Lithuania (L), Ukraine (U), Russia (R) and Italy (I). The root explants were cultivated on IM for 12 weeks. Fifteen to twenty root explants were taken from each seedling, and 24–30 seedlings were assessed per population. Regeneration percentages of the root explants were determined for every seedling and then percentage data were sorted in the following classes: n = 0, 0 < n ≤ 20%, 20 < n ≤ 40%, 40 < n ≤ 60%, 60 < n ≤ 80% and 80 < n ≤ 100%. Data represent the proportions of seedlings belonging to each class of regeneration frequencies per population.

The distribution of regeneration frequencies of the explants per seedling showed that the most responsive Sl population consisted of highly responsive seedlings, with a high number of embryogenic explants (Fig. 4). Within Sl population, even 82% of seedlings had 80–100% of regenerating explants, whereas in populations with intermediate responsiveness (Sr, P and E), a number of seedlings (approximately 40%) exhibited an intermediate response with 20–60% of regenerating explants, and 21–28% of seedlings were irresponsive (Fig. 4). In the least responsive populations (G, L and U), the explants of 52–85% of seedlings were irresponsive, and the explants of only up to 1.3% of the seedlings regenerated SEs at the frequencies above 60% (Fig. 4). Finally, R and I seedlings were completely irresponsive (Fig. 4).

**Figure 5:**
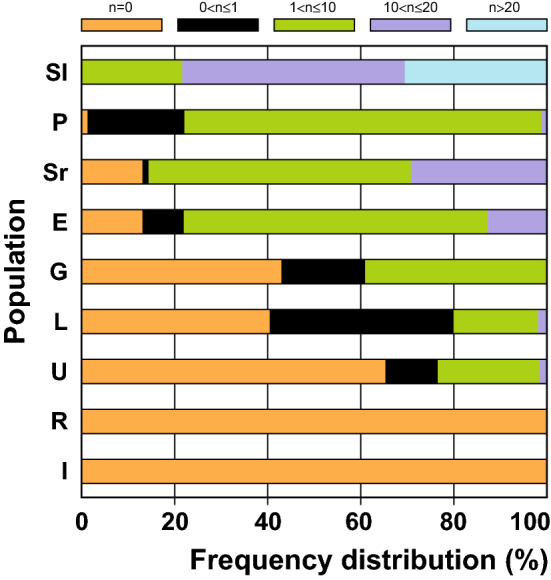
Frequency distribution of the proportion of seedlings belonging to five classes of the mean somatic embryo numbers per explant. Seedlings of nine populations of spinach cultivar Matador from Slovenia (Sl), Poland (P), Serbia (Sr), England (E), Germany (G), Lithuania (L), Ukraine (U), Russia (R) and Italy (I) were tested. The root explants were cultivated on IM for 12 weeks. Fifteen to twenty root explants were taken from each seedling, and 24–30 seedlings were assessed per population. The mean SE numbers per explant were calculated for each seedling and classified in the appropriate class: n = 0, 0 < n ≤ 1, 1 < n ≤ 10, 10 < n ≤ 20, and n > 20. Data represent the proportions of seedlings belonging to each class of the mean SE numbers per population.

The explants of Sl seedlings also regenerated the highest number of SEs: 19.6% of the seedlings regenerated 1 < n ≤ 10 SEs, while 43.5% and 27.6% of the seedlings regenerated 10 < n ≤ 20 and > 20 SEs per explant (Fig. 5). In Sr population, 54.2% and 27.9% of seedlings regenerated 1 < n ≤ 10 and 10 < n ≤ 20 SEs, respectively, while the majority of P, E and G lines (73.8%, 59.7% and 36.1%, respectively), regenerated 1–10 SEs. Only 1% of P, E and U lines and none of G and L lines regenerated 10 < n ≤ 20 SEs per explant (Fig. 5).

### Testing a sampling method of plant material using the expression of *SoRIP2* gene

**Figure 6:**
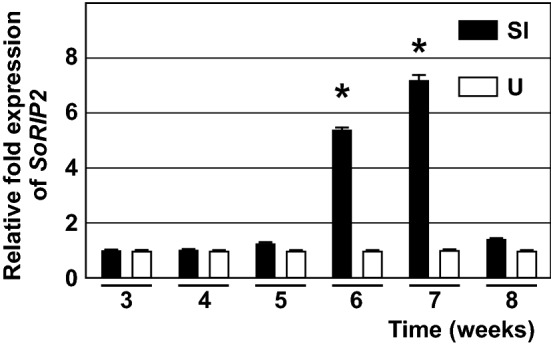
Expression profile of *SoRIP2* gene during somatic embryo induction from the root sections of Sl and U seedlings, cultivated on IM for 3–8 weeks. The expression was normalized to the expression of the α-tubulin gene, and calculated relative to the expression of the *SoRIP2* gene in the respective explants cultivated on IM for 3 weeks, using ΔΔCt method. Data represent mean values ± standard error of three independent biological samples, each with 3 technical repetitions. Values marked with asterisk are significantly different (p ≤ 0.05) from the control according to t-test for dependent samples.

The explants of Sl and U populations, with the highest and the lowest embryogenic capacity obtained in the present study, were further compared at six time points over the SE induction period using the expression of *SoRIP2* gene to estimate the level of embryogenic response. In Sl explants, the expression of *SoRIP2* increased starting from the 6th week, reaching a 7.4-fold increase in the 7th week of cultivation on IM, and then decreased to the control level. However, in U explants no significant differences in the expression pathern of *SoRIP2* were observed during the entire course of the experiment (Fig. 6).

In the tissue culture controls, which were set in parallel using identical plant material, the first SEs were observed on the Sl root explants in the 7th week of cultivation. Sl root explants regenerated SEs at the frequency of 80% ± 0, with 7.3 ± 1.0 SEs per explant, while no U explants regenerated SEs during 12-week period of cultivation.

## Discussion

The present study showed significant inter- and intra-population variability in embryogenic capacity of the root explants taken from seedlings of nine distantly located spinach populations of the same cultivar. The frequencies of seedling and explant responsiveness each varied from 0 to 100%, while the mean SE numbers varied between 0 and 14.4 per population and 0–36 per seedling. Sl population was superior to others, as the explants taken from Sl seedlings responded not only with the highest regeneration frequencies and the mean SE numbers, but also regenerated SEs earlier than the explants of other populations. In the previous studies, an earlier explant response was observed in lines predisposed for efficient somatic embryogenesis^17^, and also under optimal light conditions (LD photoperiod and light intensity of 100 µmol m^−2^ s^−1^) in comparison to unfavourable light conditions (short day, SD, photoperiod and lower or higher light intensity)^21^.

Such a high variability of regeneration capacity was also observed in many plant species^25,26^. Several studies found high variations in de novo regeneration response among ecotypes in *Arabidopsis*^18,20,27,28^. Even individuals within inbred or dihaploid lines exhibited variations in embryogenic response, indicating also significant impact of physiological status of donor plant material and environmental factors on this process^29–31^. Despite it is known for a long time that natural genetic variations may tremendously impact de novo plant regeneration^32^, and the underlying mechanism of this phenomenon has been intensively studied, it has not been elucidated to date^33^. The involvement of diverse proteins, whose genes are differentially expressed in lines with a high capacity for de novo regeneration, has been reported, including ferredoxin-nitrite reductase^34^, superoxide dismutase^35^, DELLA proteins^36^, a leucine-rich repeat receptor-like kinase^20^, thioredoxin^28^ or histone modifying enzymes^37^. Different functions of these proteins, and possible involvement of environmental factors on regulation of their expression, implicate a complex nature of the initiation of de novo regeneration, which include a network of hormone signalling and chromatin remodelling, followed by the modulation of gene expression, hormone levels, and redox homeostasis^18,20,28,33,37–40^.

Variability in embryogenic capacities observed in the present study shows that care has to be taken in choosing plant material for experimentation, because choosing more responsive individuals for one treatment and less responsive ones for another may lead to misinterpretation of the obtained results. For this reason, in a previous study^21^ we used genetically identical spinach donor plants, grown under the same conditions, to study the effect of light on SE regeneration from the root sections, and undoubtedly demonstrated that light, in terms of photoperiod and light intensity, strongly affected embryogenic capacity of the root explants of spinach, with LD conditions and light intensity of 100 µmol m^−2^ s^−1^ being optimal. By contrast, Geekiyanage et al.^41^ found bud regeneration from the cotyledonary explants of spinach cv. Longstanding Bloomsdale Dark Green grown under SD conditions to be more efficient. These discrepancies may be due to different light requirements of the root and cotyledonary explants for induction of de novo regeneration. However, nearly all differences among treatments in the study of Geekiyanage et al.^41^ were statistically insignificant, indicating that this may be due to a high variability of the plant material.

Nevertheless, it is not always possible to obtain enough plant material of the same lines for experimentation, thus we sought for a sampling method to annul variability among the plants. In order to examine the usefulness of a new sampling method of the explants, we used expression of *SoRIP2* to quantify embryogenic response of the explants, as well as a conventional counting of SEs.

The results on *SoRIP2* expression profile data were as expected and in agreement with conventional SE counting, indicating that the used plant sampling method was adequate. The expression of *SoRIP2* gene was significantly higher in Sl explants cultivated on IM for 6–7 weeks than in control, indicating the presence of globular to early cotyledonary stage SEs in these explants.

Surprisingly, both tissue culture and *SoRIP2* expression data showed that the explants taken from Sl seedlings for this experiment had as much as twice lower embryogenic capacity than was expected according to the results obtained in the previous experiment. This result indicates instability of regeneration response even in the population which was found to be superior in the present study. However, the mean SE number per explant obtained in this experiment was still almost two-fold higher than that of the second best population tested in the present study.

The highest increase in the expression of *SoRIP2* in Sl samples preceded the appearance of the first discernible (by a stereomicroscope) SEs for a week. This is also in accordance with the previous study, which showed the highest expression of *SoRIP2* in SEs at the globular stage of development, when they are embedded in the root proliferations and could be hardly observed even by a stereomicroscope^24^. Delayed response of the explants chosen for *SoRIP2* expression in the present study was another indicator of lower embryogenic capacity of these seedlings.

The present study strongly indicates that the variability of plant material must not be overlooked, because it may significantly affect the interpretation of the results. Also, the sampling method used in the present study was appropriate, thus also proving to be useful in the case when only seedlings of unknown embryogenic capacities are available as plant material. However, we recommend testing each seed lot in a preliminary study before setting up any experiments on somatic embryo induction in spinach. Using this approach, we were able to successfully study synergistic effect of light and gibberellins on somatic embryogenesis in spinach^42^.

## Conclusions

To conclude, a high variability of embryogenic capacity was observed both within and among the nine selected populations of spinach cultivar “Matador”. The cause of this variability is presently unknown and further experiments are needed to clarify this point. Among the populations tested, the explants taken from Sl seedlings exhibited the highest embryogenic capacity because they responded with high frequency of regeneration, producing high number of SEs. However, further testing of a new seed lot, by both conventional SE counting and using the expression of the marker gene, specifically expressed in the globular to early cotyledonary explants, showed lesser embryogenic capacity, indicating that this trait can vary from lot to lot, thus suggesting the necessity of preliminary testing of the plant material at the beginning of any research. A method for explant sampling was proposed, which proved to be adequate in the present study.

## Materials and methods

### Basal and induction medium content

The basal medium (BM) contained full strength macro and micro salts according to Murashige and Skoog^43^, 20 g/l sucrose, 100 mg/l myo-inositol, 2 mg/l thiamine, 2 mg/l pyridoxine, 5 mg/l nicotinic acid and 2 mg/l adenine. Somatic embryo (SE) induction medium (IM) contained BM supplemented with 20 μM α-naphthaleneacetic acid (NAA) and 5 μM gibberellic acid (GA_3_). GA_3_ was dissolved in absolute ethanol, sterilized by filter-sterilization (0.22 μm, Merck Millipore, Billerica, MA, USA) and added to the sterilized medium cooled to approximately 40 °C. The pH was adjusted to 5.5 before media sterilization. The media were gelled with 0.7% agar and sterilized at 114 °C (80 kPa) for 25 min.

### Plant material

Seeds of spinach cultivar Matador, obtained from nine European seed companies, located in Slovenia (Sl), Poland (P), Serbia (Sr), England (E), Germany (G), Lithuania (L), Ukraine (U), Russia (R) and Italy (I) were used in the present study (Supplementary Table S1). The seeds were produced in the same season and were stored in the same dry place at room temperature after arrival to our laboratory.

Seedlings derived from seeds of each seed company were considered a population. The seeds were sterilized and germinated as was described previously^17^. Briefly, the seeds were washed up with running tap water and a commercial detergent (Fairy, Procter and Gamble Co.), surface sterilized in a 30% bleach solution (4% NaClO) for 30 min, followed by 15% bleach solution for 15 min and then rinsed three times with sterile distilled water, blotted dry on a sterile filter-paper and planted in 90-mm Petri-dishes containing plant growth regulator (PGR)-free BM for germination. Seedlings with fully developed 5 leaves and branched root system were used as a donor material of the root explants.

### Regeneration procedure and culture conditions

For induction of somatic embryogenesis, 1-cm-long apical fragments of the lateral roots were isolated and cultivated on IM. The root explants were taken from 24–30 seedlings, randomly chosen from each seed population using three seed lots. Each seedling was considered an individual line. From each seedling, 15–20 root explants were taken and cultivated in Petri-dishes (5 explants per dish), thus 400–500 root explants were cultured per population. The explants were subcultured on fresh IM at 4-week intervals over a 12-week period. SEs were counted with the aid of a stereomicroscope at the end of each subculture and then removed from the root explants.

All cultures were exposed to a long-day (16 h light and 8 h dark) photoperiod and were maintained under diffuse light provided by cool white fluorescent tubes, with a photosynthetic photon flux density of 100 μmol m^−2^ s^−1^ at 25 ± 2 °C.

### Recordings and data analysis

In the present study, a completely randomised design was used for culture placement. For transforming data to normality, percentage data were subjected to angular and SE number data to square root transformation prior to analyses. Kolmogorov–Smirnov test was used for testing normal distribution fitting of the data. The mean values were back-transformed for presentation. In order to assess the significance of population and individual seedlings effect on the embryogenic capacity, nested ANOVA was used. The ability of the explants to regenerate SEs (referred to as “embryogenic capacity” or “embryogenic response”) was evaluated through three dependent variables: the frequency of regeneration, the mean SE number and an index of SE-forming capacity (SEFC). The index of SEFC was used to assess cumulative effect of both the frequency of regeneration and the mean SE number, and was calculated as follows: SEFC = (mean SE number per explant × % of regenerating explants)/100. The effect size of population and individuals on each dependent variable was estimated using the partial Eta-squared (Pη^2^) value.

To assess the regeneration capacity of a population, the regeneration frequency, the mean SE number and the index of SEFC were calculated per seedling. A seedling was considered responsive if at least one root explant regenerated SEs. Regeneration frequency was calculated as the proportion of responsive seedlings among the total number of seedlings tested. The regeneration frequencies and the SEFC indices were calculated per biological repetition and then the mean number was calculated for each population. The regeneration frequencies were recorded on weekly bases for each of the nine populations tested. The SE number was calculated per explant for the entire 12-week cultivation period and then the means were calculated per population. Statistical significance among populations was tested by one-way ANOVA and the means were separated using Fisher’s LSD post hoc test for P ≤ 0.05.

To assess the regeneration frequency at the seedling level, regeneration frequencies and the SEFC indices were calculated per Petri-dish, while the mean SE number was calculated per explant. Regeneration percentages of root explants were determined for each seedling and then percentage data were sorted in the following classes: n = 0, 0 < n ≤ 20%, 20 < n ≤ 40%, 40 < n ≤ 60%, 60 < n ≤ 80% and 80 < n ≤ 100% and presented as portions of seedlings belonging to each regeneration frequency class. The mean SE numbers per explant were calculated for each seedling, classified in the appropriate class: n = 0, 0 < n ≤ 1, 1 < n ≤ 10, 10 < n ≤ 20, and n > 20, and the distribution frequencies of seedlings belonging to each class were presented for each seed population.

### Testing the sampling method of plant material using the expression of *SoRIP2* gene

Concerning high variability in embryogenic capacity among the seedlings, a sampling method was established in the present study in order to annul diversity of embryogenic capacity among the randomly chosen seedlings. Equal number of root explants taken from each seedling was subjected to all treatments. At least ten randomly chosen seedlings were used per each of three biological repetitions.

The sampling method was tested using the expression of a marker gene specifically expressed in somatic embryos at the early stages of development (*SoRIP2*) and conventional SE counting to estimate the embryogenic capacity of the explants. Seedlings of Sl and U populations, with the highest and the lowest embryogenic capacities, were used for this analysis. One-cm-long apical fragments of the lateral roots were isolated and cultivated on IM for 3, 4, 5, 6, 7 or 8 weeks. The root explants were harvested 4 h after the light was turned on^24^, frozen in liquid nitrogen and kept at − 80 °C until RNA isolation. Tissue culture controls for the measurement of embryogenic capacity by SE counting were prepared in parallel and used for a comparison with *SoRIP2* expression data. The control cultures were prepared for each biological repetition and maintained for 12 weeks on IM with 4-week subcultivations, as described above.

Total RNA was extracted from 150 mg of root tissue, following the procedure of Gašić et al.^44^. The obtained RNA samples were treated with DNase I (Thermo Scientific, Waltham, MA, USA) at 37 °C for 30 min, in order to remove genomic DNA contamination. First strand cDNA was synthesized in a 20-μl reaction mixture containing 1 µg of total RNA, using the High-Capacity cDNA Reverse Transcription Kit (Life Technologies).

Quantitative real-time RT-PCR (qRT-PCR) was performed in a QuantStudio 3 Real-Time PCR Systems (Applied Biosystems), in a 10-μl reaction mixture containing Maxima SYBR Green/Rox qPCR Master Mix (Thermo Scientific), 300 nM primers and 1 μl cDNA template. *SoRIP2* gene (GenBank accession number AB435547.1^45^) specific primers are given in Supplementary Table S3. Thermal cycling conditions for amplification were: initial denaturation at 95 °C for 5 min, followed by 35 cycles of denaturation at 95 °C for 30 s, annealing at 60 °C for 1 min and extension at 72 °C for 1 min.

Expression of *SoRIP2* gene was normalized to the expression of *α-TUBULIN* (*α-TUB*, GenBank accession number M21414.1^45^) and calculated relative to the expression of the appropriate control according to the ΔΔCt method^46^. Primers used for amplification of *α-TUB-*specific sequence are given in Supplementary Table S3. Root explants grown for 3 weeks on IM were used as a control. The experiment was conducted in three biological repetitions, each with three technical replicates. Statistical significance of gene expression data was tested using t-test for dependent samples.

## Supplementary information

Supplementary Information

## Data Availability

All data generated or analyzed during this study are included in this article and its Supplementary Information Files.
