# Supplementary material for: Variability in somatic embryo-forming capacity of spinach
Source: Sci Rep. 2020 Nov 9;10:19290. doi: 10.1038/s41598-020-76279-9 (PMC7652860; doi:10.1038/s41598-020-76279-9)
Supplement: Supplementary file 1 — Supplementary Information [file 41598_2020_76279_MOESM1_ESM.pdf]

Variability in somatic embryo-forming capacity of spinach

Maja Belić[1], Snežana Zdravković-Korać[1*], Branka Uzelac[1], Dušica Ćalić[1], Suzana Pavlović[2], Jelena Milojević[1]

[1]University of Belgrade, Institute for Biological Research "Siniša Stanković"- National Institute of Republic of Serbia, Department of Plant Physiology, Bulevar despota Stefana 142, 11 060 Belgrade, Serbia

[2]Institute for Vegetable Crops, Karađorđeva 71, 11420 Smederevska Palanka, Serbia

*Correspondence: Snežana Zdravković-Korać, koracs@vektor.net

Supplementary Information

Population	Seed company	Frequency of seed germination				5 leaf-stage (DAS)
		1 week	2 weeks	3 weeks	4 weeks	
England (E)	King Seeds, Colchester	45.23±0.42	57.96±0.39	71.38±1.37	73.47±1.73	23.43±0.11
Germany (G)	Kiepenkerl, Bad Marienberg	7.87±0.88	43.71±0.59	55.42±0.55	74.70±0.32	31.79±0.22
Russia (R)	Аэлита, Moskow	27.79±0.30	51.00±0.13	63.66±0.22	72.10±0.19	26.74±0.21
Slovenia (Sl)	Semenarna, Ljubljana	5.87±0.03	40.94±0.08	57.08±0.16	64.37±0.29	34.90±0.35
Lithuania (L)	Nojaus Seklos, Kaunas	64.42±0.47	84.42±0.15	100±0	100±0	24.70±0.08
Ukraine (U)	Nikitovka Seeds, Slowjansk	7.94±0.58	33.51±0.43	55.05±0.2	74.15±0.16	24.62±0.09
Serbia (Sr)	Seme Semena, Belgrade	32.68±0.22	56.28±0.67	88.58±0.47	90.40±0.25	22.29±0.07
Poland (P)	W. Legutka, Jutrosin	57.05±0.12	72.22±0.13	83.82±0.05	87.09±0.06	33.33±0.21
Italy (I)	Florsilva Ansaloni, Bologna	3.68±0.62	24.40±0.30	51.37±0.45	53.00±0.03	27.08±0.17

Supplementary Table S1. The rates of seed germination and seedling development for seeds obtained from nine European seed companies. Seeds were surface sterilized and planted in 90-mm Petri dishes containing plant growth regulator-free medium for germination. Five replicates (Petri-dishes), each with 20 seeds, were prepared per seed population. Time needed for seedlings to reach 5-leaf stage of development (days after sowing - DAS) was recorded since the seedlings at this stage of development were used as a donor material of the root explants. Data in the table represent means ± standard deviation.

Source of variation	SS	*df*	MS	F	p	Partial η^2
**Regeneration frequency**						
**Intercept**	143.5406	1	143.5406	2041.850	0.00	0.824374
**Population**	109.8631	8	13.7329	195.349	0.00	0.782260
**Individuals**	66.4884	225	0.2955	4.204	0.00	0.684963
**Error**	30.5802	435	0.0703			
**Mean SE number**						
**Intercept**	3130.625	1	3130.625	2629.858	0.00	0.457139
**Population**	3706.639	8	463.330	389.217	0.00	0.499257
**Individuals**	2241.246	225	9.961	8.368	0.00	0.376116
**Error**	3717.670	3123	1.190			
**SEFC**						
**Intercept**	6555.16	1	6555.165	591.1402	0.00	0.576081
**Population**	13214.42	8	1651.802	148.9584	0.00	0.732582
**Individuals**	10660.64	225	47.381	4.2728	0.00	0.688478
**Error**	4823.72	435	11.089			

Supplementary Table S2. Summary of Nested ANOVA showing inter- and intra-population variability of the regeneration frequency, the mean SE number and the somatic embryo-forming capacity (SEFC) of the root explants derived from seeds obtained from nine European seed companies. The root explants were cultivated on IM for 12 weeks. (The data refer to data shown on Supplementary Fig.S1.). SS - Sum of squares, df - degrees of freedom, MS - Mean Square, η^2 – Eta-squared

Gene	Primer sequence	Amplicon length	Source reference
SoRIP2	F: 5'- ACCTTCCTAAGTGACATACG -3'	629 bp	[24]
	R: 5'- GCATATACTAACGCAATTGG -3'		
α-TUB	F: 5'- ACCCTCGGATCCACTTCATG -3'	585 bp	[24]
	R: 5'- AGGGCAGCAAGATCCTCACG -		

Supplementary Table S3. Sequence of primers used for amplification of *SoRIP2* and *α-TUB* sequences. F- forward primer, R-reverse primer

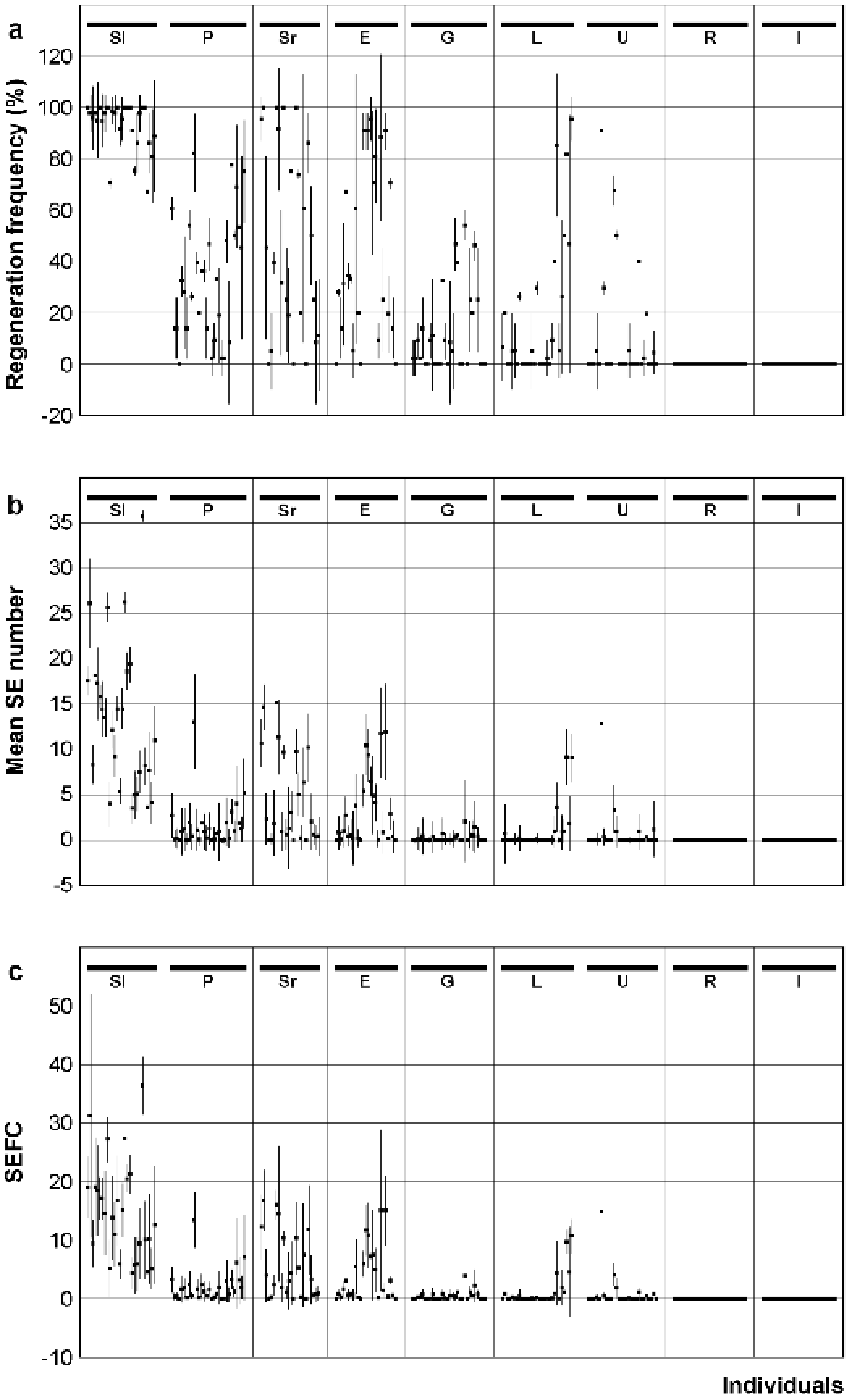
a
b
c
Sl
P
Sr
E
G
L
U
R
I
Regeneration frequency (%)
Mean SE number
SEFC
Individuals
120
100
80
60
40
20
0
-20
35
30
25
20
15
10
5
0
-5
50
40
30
20
10
0
-10

Supplementary Figure 1. Inter- and intra-population variability of embryogenic capacity in spinach. **(a)** The regeneration frequency, **(b)** the mean SE number per explant and **(c)** the index of somatic embryo-forming capacity (SEFC). Seedling populations of spinach cultivar Matador were derived from seeds obtained from nine European seed companies, located in: Slovenia (Sl), Poland (P), Serbia (Sr), England (E), Germany (G), Lithuania (L), Ukraine (U), Russia (R) and Italy (I). Fifteen to twenty root explants were taken from each seedling and cultivated in Petri-dishes (5 explants per dish) on IM for 12 weeks. Twenty-four to thirty seedlings were assessed per population. The regeneration frequency and the SEFC index were calculated per replicate (Petri-dish), while the mean SE number was calculated per explant, for each seedling. Data represent the mean, while the vertical bars denote standard deviation.
